# Producing and scrounging can have stabilizing effects at multiple levels of organization

**DOI:** 10.1002/ece3.6111

**Published:** 2020-02-19

**Authors:** William L. Vickery

**Affiliations:** ^1^ Département des Sciences biologiques, et Groupe de recherche en écologie du comportement Université du Québec à Montréal Montreal QC Canada

**Keywords:** adaptive dynamics, biodiversity, competition, density dependence, food chain, predation, producing, scrounging, stability

## Abstract

This study shows, for the first time, that the evolution of a simple behavior, scrounging, at the individual level can have effects on populations, food chains, and community structure. In particular, the addition of scrounging in consumer populations can allow multiple consumers to coexist while exploiting a single prey. Also, scrounging in the top predator of a tritrophic food chain can stabilize interactions between the top predator, its prey, and its prey's prey. This occurs because the payoffs to scrounging for food in a population are negative frequency dependent, allowing scroungers to invade a population and to coexist with producers at a frequency which is density‐dependent. The presence of scroungers, who do not search for resources but simply use those found by others (producers) reduces the total amount of resource acquired by the group. As scrounging increases with group size, this leads to less resource acquired per individual as the group grows. Ultimately, this limits the size of the group, its impact on its prey, and its ability to outcompete other species. These effects can promote stability and thus increase species diversity. I will further suggest that prey may alter their spatial distribution such that scrounging will be profitable among their predators thus reducing predation rate on the prey.

## Graphical abstract

Scrounging, a behaviour which can be expected to evolve when animals forage together, can impact both population dynamics and community structure. The fact that scrounging evolves under frequency‐dependent selection and has density‐dependent effects is key to stabilizing interactions with competitors and with prey. These effects can cause top‐down stabilization of a food chain and can promote species diversity.

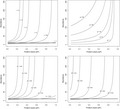

## 1 INTRODUCTION

It has long been understood that the behavior of individuals can impact the dynamics of their populations. The defense of territories limits the number of individuals who have access to resources and thus sets limits to population density (Ochiai & Susaki, 2002, Japanese goat‐antelopes; Grant & Kramer, 1990, juvenile salmonids). Cannibalism can increase mortality rates and thus reduce recruitment rates (see Wissinger, Whiteman, Denoël, Mumford, & Aubee, 2010). When individuals decide to live in groups, they can both benefit from decreased mortality through group defense and suffer losses due to intraspecific resource competition; either can result in changes to population growth rate (Clutton‐Brock et al., 1999; Courchamp, Clutton‐Brock, & Grenfell, 1999; Stephens, Sutherland, & Freckleton, 1999). The decision to switch from consuming one prey to another may or may not stabilize the predator–prey interaction (Abrams, 1999).

Here, I will analyze the effects of a social behavior (scrounging; Barnard & Sibly, 1981) not only on predator–prey interactions but also within a food chain and on interspecific competition. Scroungers exploit food sources found by others (referred to as producers). The conditions under which scrounging is expected to evolve have been established theoretically (Vickery, Giraldeau, Templeton, Kramer, & Chapman, 1992) and tested experimentally (see Giraldeau & Caraco, 2000). Specifically, scrounging should occur when foragers can detect the foraging success of others and when food is found in patches, which the producer cannot avoid sharing.

Coolen, Giraldeau, and Vickery (2007) noted that when animals scrounge they do not search for food. Thus, a group of animals in which scrounging occurs will spend less time looking for food than a group without scroungers. As a result, as scrounging frequency increases in a group total effort allocated to hunting for food will decrease and thus the total amount of food found and consumed should decrease. Further, as group size increases in the Coolen et al. (2007) model the frequency of scrounging increases producing a negative density‐dependent effect which can limit population growth and stabilize predator–prey interactions. Toyokawa (2017) showed that scrounging in a predator–prey system can overcome the destabilizing effects of the “paradox of enrichment”.

It has long been known that density‐dependent effects can stabilize population size (Verhulst, 1838). More recently, Rosenzweig and MacArthur (1963) and May (1973) have emphasized the effects of density‐dependent effects on the stability (or lack thereof) of interspecific interactions. Such effects can arise from intraspecific interference, food switching, and even coevolution of species interactions (Mougi & Nishimura, 2008; Rosenzweig, 1973). Here, I will show that the evolution of scrounging in a population can produce density dependence, which can affect food chain stability and the coexistence of competitors.

Fryxell and Lundberg (1998) proposed that individual behavior can influence community dynamics. They predicted that social behaviors (particularly direct interference and territoriality), along with systematic foraging and central‐place foraging, have the strongest stabilizing effects on predator–prey dynamics. In their model of direct interference, individuals lose foraging opportunities whenever they encounter a conspecific. As encounters increase with population density, foraging efficiency will decrease with density thus producing a stabilizing effect on density. They also argue that optimal territory size will limit the density of the foraging population while protecting their resources from excessive predation.

Here, I will show that the effects of scrounging can go far beyond population and predator–prey dynamics. A behavior which we can expect to evolve within populations can affect not only the population dynamics within a species but also food chain dynamics, interspecific competition and coexistence and, ultimately, species diversity. I will show the potential power of scrounging to stabilize predator–prey and competitive interactions by adding scrounging to simple models of these interspecific interactions which lack stability. If model dynamics are stabilized by the addition of scrounging, I will conclude that scrounging has a potential stabilizing effect.

## 2 THE MODEL

My analysis begins with the Coolen et al. (2007) model that describes the interaction between a predator species, which includes both producers and scroungers and its prey species. The predator species was divided into g groups of G individuals each. (The total population is thus N=gG.) This predator interacts with its prey under a Lotka‐Volterra predator–prey model. It is well known that, without scrounging, this parsimonious model produces only neutral stability (limit cycles) between predator and prey. Coolen et al. (2007) showed that the presence of scrounging in the predator population produces a stable predator–prey equilibrium. Furthermore, as the frequency of scrounging in the population increases, the equilibrium density of both prey and predators should also increase.

Here, I extend the Coolen et al. (2007) model by adding another level to the food chain. In the Coolen et al. (2007) model, an abundance P of predators preys on a population of R resources. The predators occur in groups (or patches), perhaps but not necessarily, because their food occurs in patches. The Coolen et al. (2007) model is as follows:

$$\text{dR}\;/\;\text{dt}\;=rR-\alpha RP\left(\frac{g}{P}+\frac{a}{F}\right)$$

$$\text{dP}\;/\;\text{dt}=P\left(e_{p}\alpha R\left(\frac{g}{P}+\frac{a}{F}\right)-\vartheta \right)$$

where r is the intrinsic rate of growth of the resources, α is the encounter rate between predators and resources, $e_{p}$ is the conversion rate of resources eaten to new predators, and ϑ is the mortality rate of predators independent of food consumption. The ratio *a*/*F* is the finder's share, the proportion of a given food patch which is consumed by the producer which finds the patch prior to the arrival of scrounging conspecifics. The term ((*g*/*P*) + (*a*/*F*)) is the proportion of each group, and thus of the whole population, which produces (searches for food) rather than scrounging. Collen et al. (2007) derive this as ((1/*G*) + (*a*/*F*)) in each group of G individuals based on the ESS frequency of producers in Vickery et al. (1992). We replace 1/*G* by the equivalent *g*/*P* so that predator density remains explicit in the model. (Thus, population growth implies increased group size.) (Note that this analysis assumes group size varies with group density as the group forages in an area of fixed size. I make the same assumption in the present analysis.)

For the current analysis, I add a trophic level, say herbivores (H), to the Coolen et al. (2007) model. In this new model, scrounging occurs only in the top predator (not in the consumption of resources by herbivores) giving:

dR/dt=rR-αRH

$$\text{dH}\;/\;\text{dt}=H\left(e\alpha R-\beta P\left(\frac{g}{P}+\frac{a}{F}\right)-\mu \right)$$

$$\text{dP}\;/\;\text{dt}=P\left(e_{p}\beta H\left(\frac{g}{P}+\frac{a}{F}\right)-\vartheta \right)$$

That is, among top predators, the producer eats a of the F items in a patch of herbivores, before the scroungers arrive and share the remaining herbivores with the producer. In cases where only a single prey is killed at any one time, this will be the proportion of the prey item eaten by the first animal at the kill prior to the arrival of others. The parameters e and $e_{p}$ are conversion efficiencies of food consumed to offspring produced. α and β are encounter rates. Finally, μ and ϑ are the mortality rates, independent of predation, for herbivores and predators, respectively.

Equilibrium abundances for these three growth equations can be found by setting them equal to zero and solving for R, H, and P. The only equilibrium providing coexistence for all three species occurs when:

$$R=\frac{\alpha \beta g\vartheta +\alpha \mu \vartheta -\mu e_{p}\beta r\left(a\;/\;F\right)}{e\alpha \left(\vartheta \alpha -e_{p}\beta r\left(a\;/\;F\right)\right)}$$

$$P=\frac{e_{p}\beta rg}{\vartheta \alpha -e_{p}\beta r\left(a/F\right)}$$

$$P=\frac{e_{p}\beta rg}{\vartheta \alpha -e_{p}\beta r\left(a\;/\;F\right)}$$

**Figure 1:**
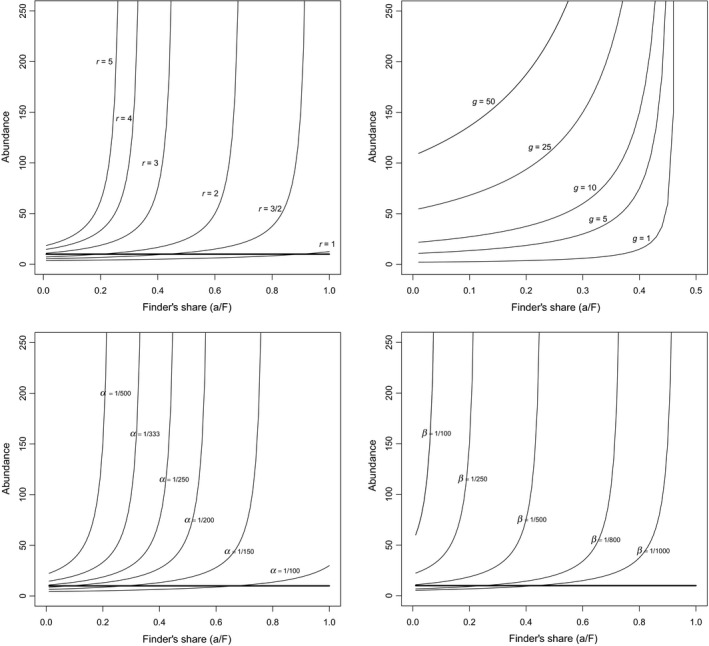
The effect of the finder's share (*a*/*F*, the proportion of food in a patch which is consumed by the finder before scroungers arrive) and other model parameters on the equilibrium abundance (number of animals in the population) of the top predator in a tritrophic food chain with scrounging in the top predator. Note that the smaller the finder's share the more scrounging will occur. Whenever the top predator has a positive equilibrium abundance, the herbivore and the resource (not shown here for the sake of simplicity) also have positive equilibria. (a) Finder's share and the growth rate (r) of the resource; (b) finder's share and the number of groups of the top predator (g); (c) finder's share and the encounter rate of herbivores with resources (∝); and (d) finder's share and the encounter rate of top predators with herbivores. Thick lines in (a), (c), and (d) indicate population sizes below which groups cannot form and thus this model will not apply. Parameter values used for these graphs are F = 50, e = 1/10, $e_{p}$=1/7, μ = 1/10, ϑ = 1/10, r = 3 (except in a), g = 5 (except in b), ∝ = 1/250 (except in c), and β = 1/500 (except in d)

This equilibrium produces coexistence only if R, H, and P are positive, which will occur when $\vartheta \alpha >e_{p}\beta r\left(a\;/\;F\right)$ and $\alpha \beta g\vartheta +\alpha \mu \vartheta >\mu e_{p}\beta r\left(a\;/\;F\right)$. Box 1 shows that any positive equilibrium among these three species will be stable. Figure 1 illustrates the effect of various model parameters on the stability of the food chain. Note that Figure 1 shows only the abundance of the top predator; however, whenever this species has a positive equilibrium in the figure, the herbivore and resources also have positive equilibria. In this figure, low levels of the Finder's advantage are equivalent to high rates of scrounging.

BOX 1 **Stability analysis for a tritrophic food chain after scrounging has invaded the top predator population**

A set of differential growth equations has a stable equilibrium if the real parts of the eigenvalues of its Jacobian matrix, evaluated at the equilibrium, are negative. For the tritrophic system with scrounging in the top predator, the Jacobian of the three growth equations evaluated at the equilibrium point is as follows:

$$J=\left[\begin{array}{ccc} 0 & -R\propto & 0\\ \mathrm{re} & 0 & -r\beta a/\left(\propto F\right)\\ 0 & e_{p}\beta \left(g+Pa/F\right) & \left(e_{p}r\beta a/\left(\propto F\right)\right)-\vartheta \end{array}\right]$$

which we can simplify to

$$J_{s}=\left[\begin{array}{ccc} 0 & -x_{12} & 0\\ x_{21} & 0 & -x_{23}\\ 0 & x_{32} & -x_{33} \end{array}\right]$$

because the structure of the matrix will determine the stability of the system. (In the structural matrix, all elements have the same sign as the corresponding full matrix; zeros remain zeros. The analysis thus depends on the direction of inter specific interactions rather than their exact value.) First note that all of the coefficients, $x_{\mathrm{ij}}$_,_ are positive when the system produces positive equilibrium abundances for all three species. (Negative signs in the matrix indicate a negative differential effect for a given component at the equilibrium.) The eigenvalues of $J_{s}$ are the roots of its characteristic polynomial:

$$\lambda^{3}+x_{33}\lambda^{2}+\left(x_{12}x_{21}+x_{23}x_{32}\right)\lambda +x_{12}x_{21}x_{33}$$

All the terms of the characteristic equation are greater than zero, which is a necessary condition for its roots to be less than zero. Routh‐Hurwitz criteria for this equation to have roots with only negative real parts are as follows: $x_{33}>0\;\text{and}\;x_{23}x_{32}>0\;\text{and}\;x_{12}x_{21}x_{33}>0$. Clearly, all three conditions are true because all the $x_{\mathrm{ij}}$ are positive. Therefore, the eigenvalues of the matrix $J_{s}$ all have negative real parts. Thus, the coexistence equilibria of this model are stable.

The stabilizing effect of scrounging can be seen by comparing the above results with an analysis of a tritrophic Lotka‐Volterra food chain without scrounging. (The model is easily obtained by replacing ((*g*/*P*) + (*a*/*F*)) by 1 in the above model.) Mathematically, this system of equations has an equilibrium at which all three species go extinct, another in which only the top predator goes extinct and a third in which resources go extinct and herbivores coexist with a negative abundance of top predators. Thus, without scrounging the model has no three‐species equilibrium; the addition of scrounging has the potential, for some parameter values, to stabilize the tritrophic interaction.

The fact that a stable equilibrium is possible shows that the presence of scrounging in the top predator species can induce stability further down the food chain. It is interesting to note that as the frequency of scrounging increases in the top predator population, the equilibrium abundance of both the top predator and the resource (but not the herbivore) decrease. On the other hand, as the frequency of scrounging decreases (i.e., finder's advantage increases in the figures), equilibrium abundances of resources and top predators increase up to a threshold value beyond which the tritrophic system becomes unstable. (This threshold can be seen, approximately, in Figure 1 as the value of the finder's share at which predator abundance increases almost vertically.)

High growth rates (r) of the resource require high scrounging frequencies in order to prevent instability on the system (Figure 1a). On the other hand, low resource growth rates allow coexistence only when the finder's advantage is high (i.e., scrounging frequency is low). When herbivores have high encounter rates with their resources (Figure 1c), they need low scrounging frequencies or else the top predator will exist in groups of fewer than two individuals—and thus, this model will not apply. On the other hand, low encounter rates between herbivore and resources will require high scrounging frequencies (Figure 1d) in order to avoid instability due to excessively high predator abundances.

When there are many predator groups, frequent scrounging will be needed in order to stabilize the system (Figure 1b). When only a few groups are present, low levels of scrounging will suffice to ensure stability but may result in low predator abundances. Efficient conversion of herbivores to predators $\left(e_{p}\right)$ has an effect similar to high encounter rate (no figure presented): A high frequency of scrounging is required to stabilize the system. Conversion efficiency of resources to herbivores has no effect on stability. Finally, as the mortality rate of herbivores $\left(\mu \right)$ increases so does the equilibrium abundance of resources but this has no effect on the abundance of top predators. As the mortality rate $\left(\vartheta \right)$ of the top predator increases, the frequency of scrounging which can be tolerated decreases. At low mortality rates, higher scrounging frequencies are necessary in order to stabilize the system.

Having shown that scrounging can have a stabilizing effect further down the food chain, I now ask if it might also affect interactions with competitors. To do this, I once again start with a Lotka‐Volterra model of predator–prey interactions with no scrounging. I add a competitor (a second predator), $N_{2}$ to the model choosing parameter values which ensure that this competitor is less efficient than the original one and thus loses in competition with the original predator, $N_{1}$ (i.e., the new competitor always goes extinct in a two‐predator/one prey Lotka‐Volterra model in the absence of scrounging). Finally, I add scrounging to the original predator in the same way as for the food chain model above. This gives the system of growth equations:

$$dR/dt=rR-\alpha RN\left(g/N+a/F\right)-\beta RN_{2}$$

$$\text{dN}\;/\;\text{dt}\;=N\left(e\alpha R\left(\text{g}\;/\;\text{N}+\text{a}\;/\;\text{F}\right)-\mu \right)$$

$$dN_{2}/dt=N_{2}\left(e_{2}\beta R-\vartheta \right)$$

where parameter definitions are similar to those in the food chain model.

This system of growth equations has an equilibrium at:

$$R=\vartheta /\left(e_{2}\beta \right)$$

$$N=\frac{e\alpha \vartheta g}{\mu e_{2}\beta -e\alpha \vartheta \left(a/F\right)}$$

$$N_{2}=\frac{re\vartheta \left(a\;/\;F\right)-r\mu e_{2}\beta +\mu e_{2}\beta }{\beta \left(e\alpha \upsilon \left(a\;/\;F\right)-\mu e_{2}\beta \right)}$$

Every set of positive equilibria for these three species is stable (Box 2). Equilibria will be positive when $\mu e_{2}\beta >e\alpha \vartheta \left(a\;/\;F\right)$ and $r\mu e_{2}\beta >\mu e_{2}\beta +re\vartheta \left(a\;/\;F\right)$.

BOX 2 **Stability analysis for multiple competitors, of which all but one has been invaded by scroungers, exploiting a single food source**

We can derive the Jacobian of the model for two competitors exploiting a single resource from the model given in the main text. At equilibrium the Jacobian has the structure

$$J_{s}=\left[\begin{array}{ccc} 0 & -x_{12} & -x_{13}\\ x_{21} & -x_{22} & 0\\ x_{31} & 0 & 0 \end{array}\right]$$

where all the *x_ij_* are positive and negative signs indicate negative components in the Jacobian matrix. The characteristic polynomial is $\lambda^{3}+x_{22}\lambda^{2}+\left(x_{12}x_{21}+x_{13}x_{31}\right)\lambda +x_{13}x_{31}x_{22}$. As with tritrophic model, it is clear that all the terms of this equation are greater than zero. Routh‐Hurwitz criteria for this equation to have roots with only negative real parts are as follows: *x*_22_ > 0 and *x*_12_ *x*_21_ > 0 and *x*_13_ *x*_13_ *x*_22_ > 0. Thus, all the eigenvalues of the system have only negative Real parts. Therefore, all positive equilibria of the model are stable.

When scrounging has invaded two of the competitors, but not the third, the Jacobian of the system of growth equations is as follows:

$$J=\left[\begin{array}{cccc} r-\propto_{1}N_{1}\left(\frac{g_{1}}{N_{1}}+\frac{a_{1}}{F}\right)-\propto_{2}N_{2}\left(\frac{g_{2}}{N_{1}}+\frac{a_{2}}{F}\right)-\propto_{3}N_{3} & -R\propto_{1}\frac{a_{1}}{F} & -R\propto_{2}\frac{a_{2}}{F} & -\propto_{3}R\\ N_{1}e_{1}\propto_{1}\left(\frac{g_{1}}{N_{1}}+\frac{a_{1}}{F}\right) & e_{1}\propto_{1}R\frac{a_{1}}{F}-\mu & 0 & 0\\ \propto_{2}N_{2}e_{2}\left(\frac{g_{2}}{N_{1}}+\frac{a_{2}}{F}\right) & 0 & e_{2}\propto_{2}R\frac{a_{2}}{F}-\vartheta & 0\\ \propto_{3}N_{3}e_{3} & 0 & 0 & \propto_{3}Re_{3}-\rho \end{array}\right]$$

We can substitute the equilibria values for population size into the matrix and reduce it to a structural matrix as before:

$J_{s}=\left[\begin{array}{cccc} 0 & -x_{12} & -x_{13} & -x_{14}\\ x_{21} & -x_{22} & 0 & 0\\ x_{31} & 0 & -x_{33} & 0\\ x_{41} & 0 & 0 & 0 \end{array}\right]$where all the $x_{\mathrm{ij}}$ are positive and negative signs in the matrix indicate a negative differential effect for a given component at the equilibrium. (Note that the bottom right element of the matrix, $\propto_{3}Re_{3}-\rho$ becomes zero at the equilibrium because, at equilibrium *R* = *ρ*/*e*_3_ *α*_3_.) The characteristic polynomial of this matrix is as follows:

$$\begin{array}{c} \lambda^{4}+\left(x_{22}+x_{33}\right)\lambda^{3}+\left(x_{22}x_{33}+x_{12}x_{21}+x_{13}x_{31}+x_{14}x_{41}\right)\lambda^{2}\\ \;+\left(x_{13}x_{31}x_{22}+x_{14}x_{41}x_{22}+x_{14}x_{41}x_{33}+x_{12}x_{21}x_{33}\right)\lambda +x_{14}x_{41}x_{22}x_{33} \end{array}$$

From the signs of the coefficients listed above, we can see that all the terms in the characteristic polynomial are positive. Routh‐Hurwitz criteria for this equation to roots with only negative real parts are as follows: $x_{14}x_{41}x_{22}x_{33}>0$ and $x_{22}+x_{33}>0$ and $\left(x_{22}^{2}x_{33}+\left(x_{12}x_{21}+x_{33}^{2}\right)x_{22}+x_{13}x_{31}x_{33}\right)/\left(x_{22}+x_{33}\right)>0$ and $(x_{13}x_{22}^{3}*x_{31}*x_{33}+\left(\left(x_{12}x_{21}+x_{13}*x_{31}\right)*x_{33}^{2}+x_{12}x_{21}\left(x_{13}*x_{31}+x_{14}*x_{41}\right)\right)*x_{22}^{2}+\left(x_{12}x_{21}x_{33}^{2}+x_{12}^{2}x_{21}^{2}+x_{14}*x_{21}*x_{41}*x_{12}+x_{13}*x_{31}*\left(x_{13}*x_{31}+x_{14}*x_{41}\right)\right)x_{22}*x_{33}+x_{13}*x_{31}*x_{33}^{2}\left(x_{12}x_{21}+x_{14}x_{41})\right)/\left(x_{22}^{2}*x_{33}+\left(x_{12}x_{21}+x_{33}^{2}\right)*x_{22}+x_{13}*x_{31}*x_{33}\right)>0$.

Because all the coefficients in each inequality are positive, the four conditions are all true.

Thus, the positive equilibrium of the three competitor system is stable.

Now that scrounging has allowed a less efficient competitor to coexist in this system, we can push the analysis one step further by asking if both these competitors incur scrounging will a third, less efficient, competitor be able to coexist with them. The system of growth equations becomes:

$$dR/dt=rR-\alpha_{1}RN_{1}\left(g_{1}/N_{1}+a_{1}/F\right)-\alpha_{2}RN_{2}\left(g_{2}/N_{2}+a_{2}/F\right)-\alpha_{3}RN_{3}$$

$$dN_{1}/dt=N_{1}\left(e_{1}\alpha_{1}R\left(g_{1}/N_{1}+a_{1}/F\right)-\mu \right)$$

$$dN_{2}/dt=N_{2}((e_{2}\alpha_{2}R(g_{2}/N_{2}\;+a_{2}/F))-\vartheta )$$

$$dN_{3}/dt=N_{3}\left(e_{3}\alpha_{3}R-\rho \right)$$

This set of equations also has an equilibrium:

$$R=\rho /e_{3}\alpha_{3}$$

$$N_{1}=\frac{\rho e_{1}\alpha_{1}g_{1}}{\mu e_{3}\alpha_{3}-\rho e_{1}\alpha_{1}\left(a_{1}/F\right)}$$

$$N_{2}=\frac{\rho e_{2}\alpha_{2}g_{2}}{\upsilon e_{3}\alpha_{3}-\rho e_{2}\alpha_{2}\left(a_{2}/F\right)}$$

$$N_{3}=\frac{r}{\alpha_{3}}-\frac{\mu \alpha_{1}g_{1}e_{3}}{\left(\mu e_{3}\alpha_{3}-\rho e_{1}\alpha_{1}\left(a_{1}/F\right)\right)}-\frac{\vartheta \alpha_{2}g_{2}e_{3}}{\left(\vartheta e_{3}\alpha_{3}-\rho e_{2}\alpha_{2}\left(a_{2}/F\right)\right)}$$

**Figure 2:**
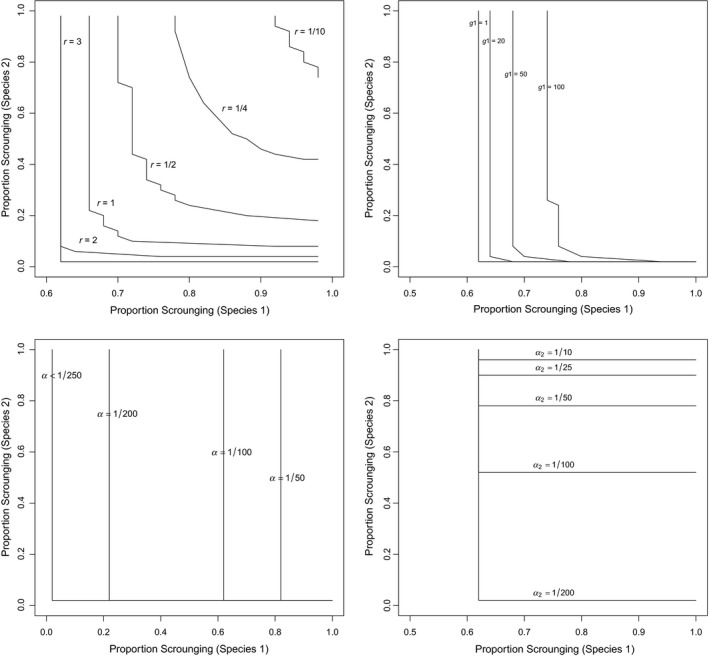
The effect of some model parameters on the proportion of scrounging necessary to allow stable coexistence of three species consuming just one resource. (Stable coexistence occurs only above and to the right of the lines in the figure.) (a) Growth rate of the resource; (b) the number of groups in the population of the most efficient consumer; (c) the encounter rate of the most efficient consumer with resources; and (d) the encounter rate of the second most efficient consumer with resources. (The previous two species both have scrounging while the least efficient species does not.) Note that the most scrounging will occur when the finder's share is smallest. (Jagged lines occur in this figure because the finder's advantages ($a_{1}$ and $a_{2}$) take on only integer values in this analysis.) Parameter values used for these graphs are F = 50, $e_{1}$ = 1/10, $e_{2}$ = 1/10, $e_{3}$ = 1/7, μ = 1/10, ϑ = 1/8, ρ = 1/7, r = 3 (except in a), $g_{1}$ = 5 (except in b), $g_{2}$ = 7, $\propto_{1}$ = 1/100 (except in c), $\propto_{2}$ = 1/200 (except in d), and $\propto_{3}$ = 1/250. (Note that species 1 forages more efficiently than species 2 which in turn forages more efficiently than species 3)

This equilibrium involves positive abundances for all four species provided that $\mu e_{3}\alpha_{3}>\rho e_{1}\alpha_{1}\left(a_{1}/F\right)$ and $\nu e_{3}\alpha_{3}>\rho e_{2}\alpha_{2}\left(a_{2}/F\right)$ and r is sufficiently large to outweigh the negative impact of the second and third terms in the equation for the equilibrium value of $N_{3}$. Once again, every equilibrium in which all four species have positive abundances is stable (Box 2). The effect of several model parameters on stability is illustrated in Figure 2. In this figure, high rates of scrounging are equivalent to low levels of the Finder's advantage ($\alpha_{1}$ or $\alpha_{2}$).

Low resource growth rate may be insufficient to support all three consumers regardless of scrounging (Figure 2a). Even high rates of resource growth will require substantial scrounging in both of the more efficient consumers in order to allow a third, less efficient consumer to invade the system and coexist in a stable equilibrium. The number of groups in each consumer species ($g_{1}$ shown in Figure 2b and $g_{2}$ not shown) has a smaller effect on coexistence with fewer groups in a given consumer species allowing lower frequencies of scrounging in that species.

Increasing encounter rates between a consumer and its resources requires higher scrounging frequency (in the given species) to stabilize the system. This result holds for the two most efficient foragers (those which experience scrounging; Figure 2c,d). For the third species (least efficient but without scrounging), low encounter rates with resources require the highest rates of scrounging in the other two species in order to maintain a stable equilibrium (not shown).

Analysis of this model suggests that high levels of conversion efficiency in either of the two species containing scrounging will require high frequencies of scrounging in that species whereas low conversion efficiency in the nonscrounging species will require high scrounging frequencies in both of the other species in order to maintain a stable equilibrium. Similarly, low mortality rates in the species with scrounging or high mortality in the species without it will require high scrounging frequencies in order to maintain a stable equilibrium.

## 3 DISCUSSION

These models show that if scrounging invades a top predator population it can generate stability in this predator's food chain and it can allow weaker competitors to coexist with the predator. Intraspecific scrounging in more than one competing predator can allow multiple competitors to coexist. This suggests that the evolution of a behavior (in this case scrounging) within a population of one species can have a substantial impact on the diversity of species in a community.

The point here is that evolution at the individual level within a population has the potential to stabilize a population, a food chain, or a community. I do not contend that scrounging will always stabilize population dynamics, food chains, and competitor interactions. In fact, the stability analyses show that only certain parameter values will produce stability. In other cases, the arrival of scrounging in a population might, when scrounging becomes too prevalent, actually push the population to extinction. Other factors (food abundance, variations in weather, predators and parasites, for instance) may have stronger effects which can completely obscure the effects of scrounging. In any case, the presence of scrounging in a population will decrease the growth rate of a population as the abundance of that species increases because increases in group size will increase the proportion of scroungers in the species and thus decrease the rate at which food is found. (It should be noted that if population growth results only in more groups without changing group size, stabilizing effects will not occur.)

An alternate to my model might be a case in which producers compensate for the food lost to scroungers by foraging over a longer time period. They could, in theory, forage until they consumed as much food as they would have if no scroungers were present. However, in this case the producers and the scroungers would be exposed to predators longer and would expend more energy while they are foraging. The resulting costs would then decrease survival, and probably birth rates, in the population resulting in density‐dependent effects and the same conclusions which I have drawn from the above model.

I use exponential growth and Lotka‐Volterra models as a base line in my analysis not because I believe that they represent exact models of intraspecific and interspecific dynamics in nature but rather because they are simple models which capture the essentials of population growth (animals are born and later die: exponential growth; the amount of food consumed influences birth and death rates as do losses to predators: Lotka‐Volterra.) These models are parsimonious and not inherently stable. Thus, they serve as a convenient null model to which one can add effects which may stabilize, or not, the dynamics being studied. The neutral stability of the two‐species Lotka‐Volterra predator–prey model is particularly useful for determining whether an added factor increases or decreases stability in the interaction. While the tritrophic Lotka‐Volterra model has no three‐species equilibria, the fact the scrounging can produce a stable equilibrium demonstrates the power of the density dependence produced by scrounging.

Others (Mougi & Nishimura, 2008; Toyokawa, 2017) have eschewed the use of simple Lotka‐Volterra models in favor of Rosenzweig and MacArthur’s (1963) model including a density‐dependent factor. This permits comparison with the “Paradox of enrichment.” I do not use this approach because it relies on an arbitrary density‐dependent term. I prefer to let the effect of scrounging produce the density‐dependent effect in the model, thus showing that scrounging can stabilize an otherwise unstable system.

I test for equilibrium based on the structure of the Jacobian matrix (i.e., the community matrix) rather than using the exact values of the Jacobian of my model. The latter would show that the particular model which I have adopted produces stability. By analyzing the matrix structure (noting only whether components are positive, negative, or zero), I show that not only my model, but also any community producing this matrix structure, will be stable, a more general conclusion.

My analysis shows that scrounging has the possibility of stabilizing a tritrophic interaction. It should be easy to show that the stabilizing effect in the food chain can be passed on to lower levels. Thus, I surmise that scrounging in a top predator species can have a stabilizing effect, which passes all the way down the food chain. It should be noted that this effect does not pass up the food chain. When I introduced scrounging in the intermediate predator population (results not shown here), the dynamics of its interaction with the top predator were not stabilized because of a lack of negative feedback on prey consumption by the top predator. I also suggest that if scrounging allows three competing species to coexist the effect can be extended to four or more competitors provided that scrounging invades the most efficient (in terms of finding food) competitors.

My analysis is similar to that of Fryxell and Lundberg (1998) in that I compare Lotka‐Volterra models, which include social foraging with those which do not. Like them, I conclude that stabilizing effects are present when the former produce stable dynamics and the latter do not. My analysis differs from theirs in that I add a third trophic level (thus allowing the stabilizing effects to be passed down the food chain) and I study the coexistence of multiple competitors consuming the same prey. In addition, I use a “Darwinian Dynamics” approach (Vincent & Brown, 2005) to the analysis in which behavioral frequencies and population size vary simultaneously. Fryxell and Lundberg (1998) assumed that behavioral frequencies would come to a stable equilibrium, which they then inserted in their Lotka‐Volterra model. It is interesting to note that, despite these differences, my analysis supports their conclusion that social behavior can have a strong stabilizing effect.

Gil, Hein, Spiegel, Baskett, and Sih (2018) showed that the use of social information can affect population stability and the coexistence of competitors. They found that social information can produce instabilities by causing “Allee” effects while in other cases sharing social information can promote species coexistence. My analysis differs from Gil et al. (2018) in that I deal not only with sharing of information (scroungers use information from producers to find food patches) but also with the sharing of a limited food resource within the food patch.

While scrounging is a behavior which changes over a shorter time scale than population size, I argue that the two should be modeled together because of the feedback between them. That scrounging can persist in populations over long time periods and respond to population size has been shown by Aplin and Morrand‐Ferron (2017).

While most studies of scrounging have focused on birds, this behavior is likely much more widespread. Harten et al. (2018) observed long‐term producer–scrounger relationships in bats. Phillips et al. (2018) analyzed a producer–scrounger game in coho salmon. I have observed scrounging among solitary Sciurids (pers. obs.) such as Noth American red squirrels. Dumke, Herberstein, and Schneider (2016) showed that scrounging frequency increases with group size in socially foraging spiders, *Australomisidia ergandros.* Recently, Kimmel, Gerlee, Brown, and Altrock (2019) have suggested that producer–scrounger type dynamics can be used in the analysis of public goods games, which they relate to the growth of cellular populations including cancers. I suggest that scrounging may occur whenever individuals forage in close proximity to one another. We should thus expect the effects of scrounging on population and community dynamics may be widespread. We should also expect that behaviors similar to scrounging, such as kleptoparasitism, will have similar stabilizing effects on populations and communities because they replace time spent searching for food with time spent taking food from others.

When scrounging stabilizes food chains or allows competitors to coexist, it may add biodiversity to ecological communities. The added competitors are, for the purposes of this analysis, functional equivalents of the species which was first invaded by scroungers. This redundancy may provide a replacement consumer in the food chain in cases where the first species becomes rare or extinct. Alternately, it may provide an alternate pathway in the food chain in cases where the additional competitors are eaten by predators which do not attack the original species. Further, in the three consumer model, when scrounging increases in the most efficient consumer it produces a smaller equilibrium abundance in that species and larger equilibrium abundance in the species which has no scrounging. One might say that scrounging “makes room” for additional competitors. This effect may have far‐reaching consequences in a food web. This is an example of how group phenotypic (Farine, Montiglio, & Spiegel, 2015; Valdivinos, Ramos‐Jiliberto, Garay‐Narváez, Urbani, & Dunne, 2010) or genetic (Genung et al., 2011) composition can involve feedback loops with community structure.

It is possible that other behaviors which can evolve within a population can have wide‐ranging effects. Vickery and Brown (2009) showed that when spite invades a population its presence can have a stabilizing effect on interactions between the population and its food supply. I have used models similar to those shown above to evaluate the possibility that this will allow additional competitors to coexist with the species which has been invaded by spite. No such coexistence is possible in my analysis of spite. Thus, I conclude that not all social behaviors have the stabilizing potential that scrounging has.

This analysis is relevant to a long‐standing ecological theme. The presence of scrounging within different species has the potential to explain the coexistence of multiple similar species within a community (Hutchinson, 1961). (I do not propose that this is the only possible explanation.) Further, scrounging may produce what Wynne‐Edwards (1965) called “self‐regulation.” In the case of scrounging, evolutionary forces within a population can lead to self‐regulation of population size without requiring “…an initiative taken by the animals themselves; …” to control their abundance. (Again, scrounging is not the only factor which can cause this apparent “self‐regulation”—see Rosenzweig, 1973.)

By looking at the results of my analyses from the point of view of the resources rather than the consumers, we can see how the spatial distribution of resources might influence diversity among consumer species. As resource patch size grows larger predator groups may be attracted to the patch. This will favor increased scrounging among predators. This increased scrounging results in lower resource consumption, another possible advantage to living in a group (it is not a dilution effect but rather an induced reduction in predator efficiency). The reduction in resource consumption will contribute stability to the interaction between predators and resources.

Overall, my analysis suggests that scrounging, a social foraging adaptation, can have a stabilizing effect on interactions between the species in which it occurs and both its prey and competitors. Further, the stabilizing effect can be passed down through the food chain and may even induce distributional changes among prey which can feed back to increase scrounging frequencies in the consumer species.

## Data Availability

No new data are used in this paper, which is based entirely on a theoretical model. Statement of authorship: I am the only author of this paper which is an extension of previous work with several colleagues, who have contributed helpful comments to this paper.
